# Fast 3D-MRSI using sparse acquisition and 4D compressed sensing reconstruction

**DOI:** 10.1007/s10334-025-01301-y

**Published:** 2025-11-01

**Authors:** Jian-Xiong Wang

**Affiliations:** Department of Radiology, University of Alabama at Birmingham, 619 19th Street South, Birmingham, AL 35233 USA

**Keywords:** Compressed Sensing, Magnetic Resonance Spectroscopic Imaging (MRSI), Hyperpolarization

## Abstract

**Background:**

Magnetic Resonance Spectroscopic Imaging (MRSI), also known as Chemical Shift Imaging (CSI), is a pivotal tool in both clinical and preclinical metabolic research. Traditional MRSI offers high sensitivity to weak metabolites and covers a wide spectral bandwidth. However, the large number of RF excitations required for fully sampled 3D-MRSI acquisitions renders it impractical for hyperpolarized (HP) MRI applications, especially given the rapid signal decay and non-renewable magnetization of HP agents such as [1-^13^C]pyruvate.

**Purpose:**

This study aims to develop and validate an accelerated MRSI method that can preserve broad spectral bandwidth and weak metabolite detectability without aliasing, overcoming limitations of fast MRSI techniques such as echo-planar spectroscopic imaging (EPSI), which typically cause narrower spectral bandwidth and can suffer from spectral aliasing.

**Methods:**

We implemented a sparsely sampled 3D-MRSI pulse sequence on an MRI scanner, acquiring data with large reduction ratios. A 4D compressed sensing (CS) reconstruction algorithm was developed to recover high-resolution spectroscopic data from undersampled measurements. The algorithm jointly reconstructs the three spatial dimensions and the frequency dimension, leveraging sparsity priors and iterative conjugate gradient optimization. The in vivo experiments were performed on a GE 3 T clinical MRI scanner (GE MR750W) using hyperpolarized [1-^13^C]pyruvate in one rat, with two acquisitions (*R* = 8 and *R* = 16) performed sequentially.

**Results:**

Our method achieved high-quality reconstructions even at acceleration factors of *R =* 16 and *R* = 32, corresponding to 6.25 and 3.125% sampling, respectively. The normalized root-mean-square error (nRMSE) and structural similarity index (SSIM) remained low (nRMSE < 4 × 10^–3^, SSIM > 0.95) even at high undersampling rates. In vivo experiments using hyperpolarized [1-^13^C]pyruvate in rat kidneys demonstrated the ability to resolve lactate, alanine, pyruvate, and bicarbonate distributions with high spatial and spectral fidelity.

**Conclusion:**

The integration of sparse MRSI acquisition and 4D-CS reconstruction enables rapid, high-fidelity MRSI with HP ^13^C-MRSI. This approach reduces acquisition time by up to 32-fold, facilitating dynamic metabolic studies and improving feasibility for routine preclinical and future clinical use.

**Supplementary Information:**

The online version contains supplementary material available at 10.1007/s10334-025-01301-y.

## Introduction

Imaging metabolism in real time holds the potential to revolutionize how we diagnose, monitor, and understand disease. While conventional MRI reveals anatomical structures, Magnetic Resonance Spectroscopic Imaging (MRSI), also known as Chemical Shift Imaging (CSI), captures the molecular fingerprints of tissue biochemistry by mapping the spatial distribution of metabolites. In its most advanced form, 3D-MRSI samples three spatial dimensions (kx, ky, kz) alongside the spectral time domain, producing a rich four-dimensional (4D) dataset that marries spatial localization with detailed chemical insight.

A key strength of MRSI lies in its ability to resolve metabolites with weak signals or closely spaced resonances, particularly when using a wide spectral bandwidth, providing high spectral resolution. This makes MRSI well suited for detailed metabolic profiling.

However, achieving high-resolution 3D-MRSI typically requires dense sampling in both spatial and spectral dimensions, which leads to long acquisition times and numerous excitations. While this is already a practical challenge in the conventional studies, it becomes prohibitive for hyperpolarized (HP) ^13^C MRI. HP techniques generate transient, non-renewable magnetization that decays rapidly due to the short T1 relaxation time. Each excitation irreversibly consumes signal, leaving only a brief window for data acquisition. Therefore, sparse sampling is not just desirable; it is essential.

In ^13^C-MRSI, particularly with hyperpolarized tracers, a broader spectral bandwidth is often required to capture multiple metabolites, and the transient nature of the magnetization necessitates rapid acquisition. These factors make ^13^C-MRSI especially well suited for CS-based acceleration. Existing acceleration strategies, such as echo-planar spectroscopic imaging, can shorten acquisition time, but this comes at the cost of reduced spectral bandwidth, since the dwell time increases to at least the normal dwell time multiplied by the reduction factor (Δt × *R*). These methods often require an additional unaliasing procedure to separate overlapping signals, an extra step that can complicate the reconstruction process and potentially degrade spectral quality [1, 2]. To overcome these limitations, we present a novel 4D compressed sensing (CS) framework that treats the entire kx-ky-kz-MRS dataset as a unified, inseparable whole. This marks a fundamental departure from conventional slice-wise or dimension-specific CS strategies. Rather than fragmenting the reconstruction process, our approach applies 4D wavelet transforms and 4D total variation (TV) regularization across the entire dataset, exploiting the natural spatio-spectral coherence of the signal. Every operation, from sparsity enforcement to reconstruction, is embedded in the full 4D structure.

This unified framework enables highly undersampled acquisition without sacrificing spectral bandwidth or spatial resolution. It is specifically tailored to the constraints of HP ^13^C-MRSI, where rapid, high-fidelity reconstruction is not just advantageous but mandatory. Unlike emerging AI- or machine learning (ML)-based reconstruction methods, which demand extensive and diverse training datasets, our model-based CS approach works directly with the acquired data. This eliminates dependence on hard-to-obtain training samples and ensures reproducibility across varied anatomical and metabolic conditions.

Building on our prior work using 3D-CS in 2D-MRSI (kx-ky-MRS) [3], we now extend the framework into full four-dimensional space. The result is a 4D-CS method that achieves unprecedented acceleration, while retaining the rich spatial and spectral resolution needed for real-time MRSI. By enforcing sparsity holistically across all dimensions, we resolve the long-standing trade-off between acquisition speed and metabolic fidelity.

Ultimately, this work addresses the central challenge of HP ^13^C-MRSI: how to capture detailed metabolic information within the fleeting window of hyperpolarization. Our 4D-CS framework transforms 3D-MRSI from a technical bottleneck into a practical, high-performance tool, advancing real-time MRSI closer to clinical reality. By uniting speed and precision, we open new frontiers in probing disease mechanisms, monitoring therapy, and guiding personalized medicine.

## Methodology

**Fig. 1:**
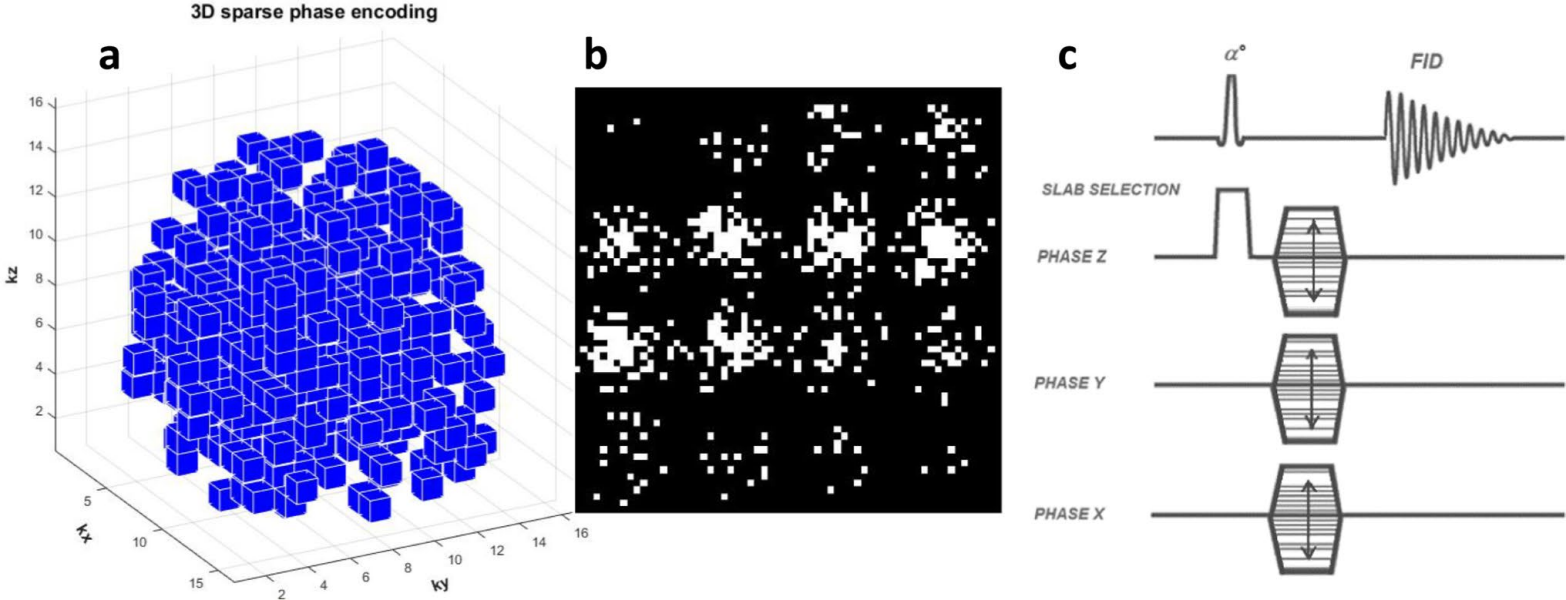
Sparsely encoded pattern in kx-ky-kz space with *N* = 16 and *R* = 8 reduction rate, resulting in a 12.5% sampling ratio. **a** Pattern in 3D, with blue voxels indicating sampled data, **b** same pattern in 16 slices with white voxels indicating sampled data, and **c** symbolic RF sequence with certain phase encodings omitted
A.2 RF sequence design and implementation
  The RF sequence, designed for hyperpolarized ^13^C spectroscopic imaging, does not require water or fat suppression, which is typically necessary in proton-based MRSI. The custom sequence (Fig. 1c) was implemented on a GE 3 T clinical MRI system (General Electric, Waukesha, WI, USA) and supports built-in 3D sparse phase-encoding acquisition for isotropic grid sizes *N* = 8 and *N* = 16, along with selectable reduction rates *R* = 1, 4, 8, 16, and 32.
  For a full grid of *N* = 16, a conventional scan would require 16^3^ = 4096 excitations. In contrast, using a reduction factor of *R* = 16, the number of acquired phase encodes is reduced to only 256, drastically shortening acquisition time while preserving sufficient signal fidelity for hyperpolarized imaging.
  Due to the non-renewable nature of hyperpolarized magnetization, small flip angles (typically 5° or less) are employed to maximize signal efficiency over multiple excitations. Phase encoding is ordered centripetally, from the center of k-space outward, using a shell-by-shell mechanism to ensure that high-SNR central data are collected early in the acquisition.
  Following acquisition, the scanner outputs a 3D complex-valued dataset of size N^3^, where acquired data points are filled with FIDs and unacquired points are assigned zeros. This structure prepares data for compressed sensing reconstruction.
A.3 Variable flip angle implementation
  An optional Variable Flip Angle (VFA) module is integrated into the RF sequence to counteract signal decay due to T₁ relaxation and repeated RF pulses. The VFA scheme progressively increases the flip angle across the scan to stabilize the signal. The flip angle for the nth excitation is computed as follows [4, 5]:
  2 $$\theta (n)={cos}^{-1}\sqrt{\frac{{E}_{1}^{2}- {E}_{1}^{2\left(N-n+1\right)}}{1-{E}_{1}^{2\left(N-n+1\right)}}}$$
  $${E}_{1}=exp\left(-\frac{TR}{{T}_{1}}\right),$$
  where *N* is the total number of excitations, TR is the repetition time, and *T*_1_ is the longitudinal relaxation time of the hyperpolarized media. This equation ensures that the last excitation ends with a flip angle approaching 90°, fully utilizing the remaining magnetization. For example, using TR = 125 ms and *T*_1_ = 30 s, the first excitation flip angle is calculated as 5.57° for *N* = 256 and 5.26° for *N* = 512, as shown in Fig. 2.

This section outlines the complete methodological framework developed for accelerated 4D ^13^C-MRSI using sparse 3D-MRSI acquisition and compressed sensing (CS) reconstruction. The approach begins with the design of a variable-density, probabilistic 3D sampling pattern tailored to the spatial characteristics of hyperpolarized imaging. A dedicated pulse sequence is implemented to acquire sparsely sampled phase-encoding data in a time-efficient manner, optionally incorporating a variable flip angle (VFA) strategy to optimize signal usage over time. The acquired sparse k-space data are then reconstructed using a 4D compressed sensing algorithm that leverages both spatial and spectral sparsity priors through wavelet and total variation regularization. The reconstruction algorithm is solved via nonlinear conjugate gradient descent. Finally, the method is validated through phantom and in vivo experiments, and performance is quantitatively assessed using standard image similarity metrics. Each of these components is described in detail in the subsections that follow:

A. Sparse 3D-MRSI acquisition
A.1 Sparse sampling pattern design
  The sparse k-space sampling pattern is generated within a 3D Cartesian grid, typically configured as a cube (e.g., 16 × 16 × 16) representing the phase-encoding space. Each voxel in this 3D grid corresponds to a unique phase-encoding step, followed by a spectroscopic readout performed without applying any imaging gradients, preserving the FID signal.
  To create an efficient sparse sampling scheme, we initiate the process by generating a 3D matrix of pseudo-random numbers *R*_*i,j,k*_ uniformly distributed between 0 and 1. This matrix is compared elementwise to a 3D Gaussian distribution ${G}_{i,j,k}$ that is centered at the origin of k-space
  $${G}_{i,j,k}=exp\left(\frac{-{\left(i-{i}_{0}\right)}^{2}+{\left(j-{j}_{0}\right)}^{2}+{\left(k-{k}_{0}\right)}^{2}}{{2\sigma }^{2}}\right).$$
  Here, (i0, j0, k0) denotes the center of the k-space-cube and σ is the standard deviation controlling the width of the Gaussian kernel. Voxels are selected into the sampling mask $V_{i,j,k}$ if the corresponding random value is below the Gaussian threshold
  1 $$V_{i,j,k} = \left\{ {\begin{array}{*{20}c} {0, R_{i,j,k} \ge G_{i,j,k} + \varepsilon } \\ {1, otherwise } \\ \end{array} } \right.$$
  The additive noise term *ϵ* ensures some stochastic variation and tuning capability in the sampling density, allowing customization of overall acceleration factors. This design naturally emphasizes the central k-space, where most signal energy resides, and progressively sparsifies the peripheral regions, which contain primarily high-frequency information. To further enhance efficiency, sampling is restricted within a spherical volume embedded inside the cubic k-space, effectively eliminating the corners and enforcing isotropic compactness. An illustration of the resulting sampling pattern is shown in Fig. 1a.

**Fig. 2:**
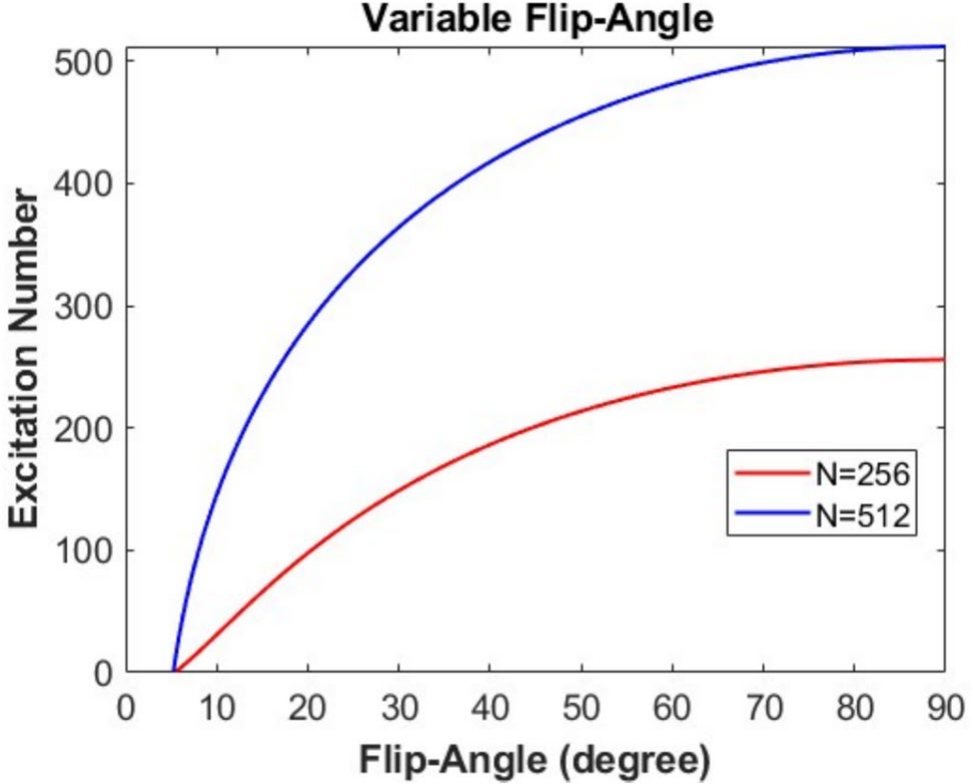
The curves show how the flip angle changes with each excitation. The *y*-axis represents the nth excitation, ranging up to 256 or 512, while the *x*-axis represents the flip angle corresponding to each excitation, ending at 90°. The first flip angle is calculated as 5.57° when the total number of excitations is 256 and 5.26° when the total number is 512
B. Reconstruction of sparse acquired data
B.1 4D compressed sensing reconstruction
  The CS reconstruction was implemented using custom-written MATLAB scripts, inspired by the SparseMRI toolbox by Michael Lustig [6]. The sparse 4D dataset (3D spatial + 1D spectral) is reconstructed using a compressed sensing (CS) approach that exploits the sparsity of MR signals in appropriate transform domains. The objective is to solve the following optimization problem [6]:
  3 $$\underset{m}{\mathrm{argmin}}|UFm-k{|}_{2}^{2}+{\uplambda }_{1}|\uppsi m{|}_{1}+{\uplambda }_{2}{\mathrm{TV}}\left(m\right).$$
  To facilitate this, the image domain m is transformed into a sparse domain using a wavelet operator *ψ*, yielding a new variable *x* = *ψm*. Optimization becomes
  4 $$\underset{x}{\mathrm{argmin}}|UF{\uppsi }^{*}x-k{|}_{2}^{2}+{\uplambda }_{1}|x{|}_{1}+{\uplambda }_{2}{\mathrm{TV}}\left({\uppsi }^{*}x\right),$$
  where $\psi$ represents the forward wavelet operator, and ${\psi }^{*}$ represents the inverse wavelet operator; the encoding matrix UF is made up of the Fourier transform F and the undersampling operator U; the variables m and k correspond to the reconstructed image and the randomly undersampled k-space data, respectively; to balance data fidelity and artifact reduction, the total variation regularization is represented as TV, and *λ*1 and *λ*2 are the associated regularization weighting parameters. The final image is reconstructed by applying the inverse wavelet transform on the variable *x*, which is represented as $m={\psi }^{*}x$. While the spectral (FID) dimension was fully sampled, the reconstruction was performed jointly across all four dimensions. The spectral dimension contributed to the sparsity structure via total variation and wavelet regularization, enhancing overall reconstruction stability. Regularization weights for total variation (TV) and sparsifying transform penalties were both set to 0.03. The maximum number of iterations was 30, with an L1 norm smoothing parameter of 1 × 10⁻^15^. Line search parameters included a maximum of 150 iterations, *α* = 0.01, *β* = 0.618, and step size reduction factor *σ* = 0.50. These values were chosen empirically from phantom data to minimize nRMSE while preventing over-smoothing.
  All operators in Eq. (4) are expressed in 4D format. The total variation of regularization *TV* is represented as follows:
  5 $$TV\left(u\right)={\sum }_{ijkl}{\left({\left({\nabla }_{1}{u}_{ijkl}\right)}^{2}+{\left({\nabla }_{2}{u}_{ijkl}\right)}^{2}+{\left({\nabla }_{3}{u}_{ijkl}\right)}^{2}+{\left({\nabla }_{4}{u}_{ijkl}\right)}^{2}\right)}^{1/2},$$
  where ∇_1_, ∇_2_, ∇_3_, and ∇_4_ denote the forward finite difference operators on three coordinates and the spectra, respectively. Reconstruction is visualized through intermediate k-space and image domain iterations. Wavelet transforms are executed in 4D using a dimension-by-dimension decomposition strategy. This allows for adaptive decomposition levels based on each axis length, which is critical in MRSI where spectral dimensions (FID) are often longer than spatial dimensions.
  For compressed sensing MRI (CS-MRI) to be effective, the underlying image data must exhibit inherent compressibility, which is typically achieved through sparse representations in a suitable transform domain, most commonly the wavelet domain [7, 8]. The discrete wavelet transform (DWT) is well established for its ability to provide such sparse representations. In this study, all wavelet operators are implemented in four dimensions to accommodate the spatial-spectral structure of the 4D MRSI dataset. Wavelet decomposition and reconstruction are performed using a dimension-by-dimension approach, whereby each dimension is adaptively transformed based on its length and signal characteristics. This adaptive strategy is particularly critical for 4D datasets with non-uniform dimensional lengths, especially in the spectral (FID) direction, ensuring optimal sparsity and reconstruction performance.
B.2 Optimization process
  A nonlinear conjugate gradient (NCG) descent algorithm with backtracking line search is employed to minimize the objective function [9]. During the optimization of Eq. (4), the variable *x* is typically initialized to zero, and iterative updates are performed using the conjugate gradient method. Specifically, each iteration computes an updated direction *dx*, and the solution is incrementally updated as ${x}_{n} = {x}_{n-1}+dx$. The iterative process is terminated when any one of the following three stopping criteria is met:
  Maximum number of iterations reached,
  Objective function change $\mathcal{F}\left({x}_{n}\right)-\mathcal{F}\left({x}_{n-1}\right)\le \varepsilon$
  Gradient step $${\Vert dx\Vert }_{2}\le \epsilon$$.
  For performance evaluation when reference data are available, two metrics are computed:
  Normalized Root-Mean-Square Error (nRMSE):
  6 $$nRMSE=\sqrt{\frac{\sum_{i=1}^{n}{\left|{x}_{i}-{y}_{i}\right|}^{2}}{n*\left(\mathit{max}\left({y}_{i}\right)-\mathit{min}\left({y}_{i}\right)\right)}.}$$
  Structural Similarity Index (SSIM):
  7 $$SSIM\left(x,y\right)=\frac{\left(2{\mu }_{x}{\mu }_{y}+{C}_{1}\right)\left(2{\sigma }_{xy}+{C}_{2}\right)}{\left({\mu }_{x}^{2}+{\mu }_{y}^{2}+{C}_{1}\right)\left({\sigma }_{x}^{2}+{\sigma }_{y}^{2}+{C}_{2}\right)},$$
  where *x* and y refer to the CS-reconstructed and fully sampled images, respectively; ${\mu }_{x}{ and \mu }_{y}, {\sigma }_{x} and {\sigma }_{y}, {\sigma }_{xy}$ represent the mean, standard deviation, and covariance of *x* and *y*. *C*_*1*_ and *C*_*2*_ are small constants used to prevent division by zero. During the validation stage, where the reference data are available, the normalized root-mean-square error (nRMSE) and the structural similarity index (SSIM) [10, 11] are computed for every iteration:
C. Validation and in vivo imaging
C.1 C.1 Phantom validation
  A custom-designed acrylic phantom (45 mm in diameter) was constructed with four internal compartments, each measuring 40 mm in length and 10–15 mm in diameter. These compartments were individually filled with thermally polarized [1-^13^C]lactate, [1-^13^C]alanine, [1-^13^C]formate, and [1-^13^C]bicarbonate. Three-dimensional chemical shift imaging (3D-MRSI) was performed using a GE 3 T clinical MRI scanner (General Electric, Waukesha, WI, USA). The acquisition protocol employed a 16 × 16 × 16 spatial phase-encoding matrix with 1024 spectral points and a spectral bandwidth of 1 kHz, covering a total imaging volume of 8 × 8 × 8 cm: resulting in an isotropic voxel size of 0.5  × 0.5 × 0.5 cm. The repetition time (TR) was set to 2 s, and the flip angle was calibrated to 90° following RF power adjustment. The fully sampled acquisition required 8192 s of total scan time. To assess the performance of compressed sensing (CS) reconstruction, undersampled datasets were retrospectively generated at various reduction factors (*R* = 2, 4, 8, 16, 32, 40, 60, 70, and 80), corresponding to 50, 25, 12.5, 6.25, 3.125, 2.5, 1.67, 1.42, and 1.25% of the full sampling density, respectively.
C.2 In vivo animal experiment
  In vivo validation of the proposed method was performed on Sprague–Dawley rats weighing between 300 and 400 g. Each rat was positioned at the isocenter of the same GE 3 T MRI system using a 75 mm dual-tuned (^1^H/^13^C) birdcage coil. An intravenous catheter was placed in the tail vein for delivery of hyperpolarized [1-^13^C]pyruvate. Anesthesia was maintained using 2–3% isoflurane, delivered via a nose cone, and physiological parameters including respiration and body temperature were continuously monitored throughout the imaging procedure. The [1-^13^C]pyruvate was polarized to approximately 20%, dissolved to a physiological temperature (37 °C) and pH (7.4), and diluted to a final concentration of 80 mM. A dose of ~ 3.0 mL was administered via tail vein injection.
  Following injection, the hyperpolarized [1-^13^C]pyruvate undergoes rapid metabolic conversion in vivo, producing [1-^13^C]lactate, [1-^13^C]alanine, and [^13^C]bicarbonate, alongside a chemical equilibrium product, [1-^13^C]pyruvate hydrate. As a result, the ^13^C spectra acquired from each voxel in the in vivo MRSI data typically display five distinct peaks corresponding to these metabolites, as shown in Fig. 7.
  3D-MRSI was performed with matrix size *N* = 16 and undersampling rates of *R* = 8 and *R* = 16, which corresponded to 512 and 256 excitations, respectively, as opposed to the 4096 excitations required for a fully sampled acquisition. Due to the impracticality of acquiring a fully encoded dataset in vivo, only undersampled acquisitions were performed. A variable flip angle scheme was employed to compensate for the progressive decay of hyperpolarized magnetization, aiming to equalize signal contributions across excitations. Spectral acquisition parameters included a bandwidth of 2.5 kHz and 256 spectral points. A 32 mm slab was prescribed to cover both kidneys, resulting in a nominal slice thickness of 2 mm. The in-plane resolution was 5 mm within an 8 cm field of view (FOV). Since no spatial filtering was applied, the effective resolution closely matched the nominal 5 mm isotropic voxel size. This provided multi-voxel coverage of the kidneys, which are ~ 1.2–1.8 cm in length in rats. Imaging was performed on a GE 3 T clinical MRI system equipped with a 75 mm dual-tuned (^1^H/^13^C) birdcage coil. The repetition time (TR) was set to 125 ms, resulting in scan durations of 58 s and 32 s for the *R* = 8 and *R* = 16 acquisitions, respectively. These two acquisitions were conducted sequentially on the same animal using two separate doses of hyperpolarized [1-^13^C]pyruvate, with an inter-scan delay of approximately 30 min to allow for metabolic clearance and signal recovery.
C.3 MR spectroscopic analysis
  In contrast to parallel imaging, compressed sensing does not attempt to interpolate or synthesize unacquired raw data. Instead, it reconstructs the final signal representations directly from undersampled measurements. In the context of MR spectroscopy, this means that CS does not recover individual missing FIDs from unacquired k-space voxels; rather, it directly reconstructs the voxel-wise spectra. Following reconstruction, each spectrum undergoes automated phase correction to minimize the dispersion (imaginary) component while preserving a well-defined absorption (real) peak suitable for quantification. Metabolite signals are then quantified and mapped voxel-wise to generate 3D-MRSI.

## Results

A. ^13^C-labeled thermal phantom imaging

**Fig. 3:**
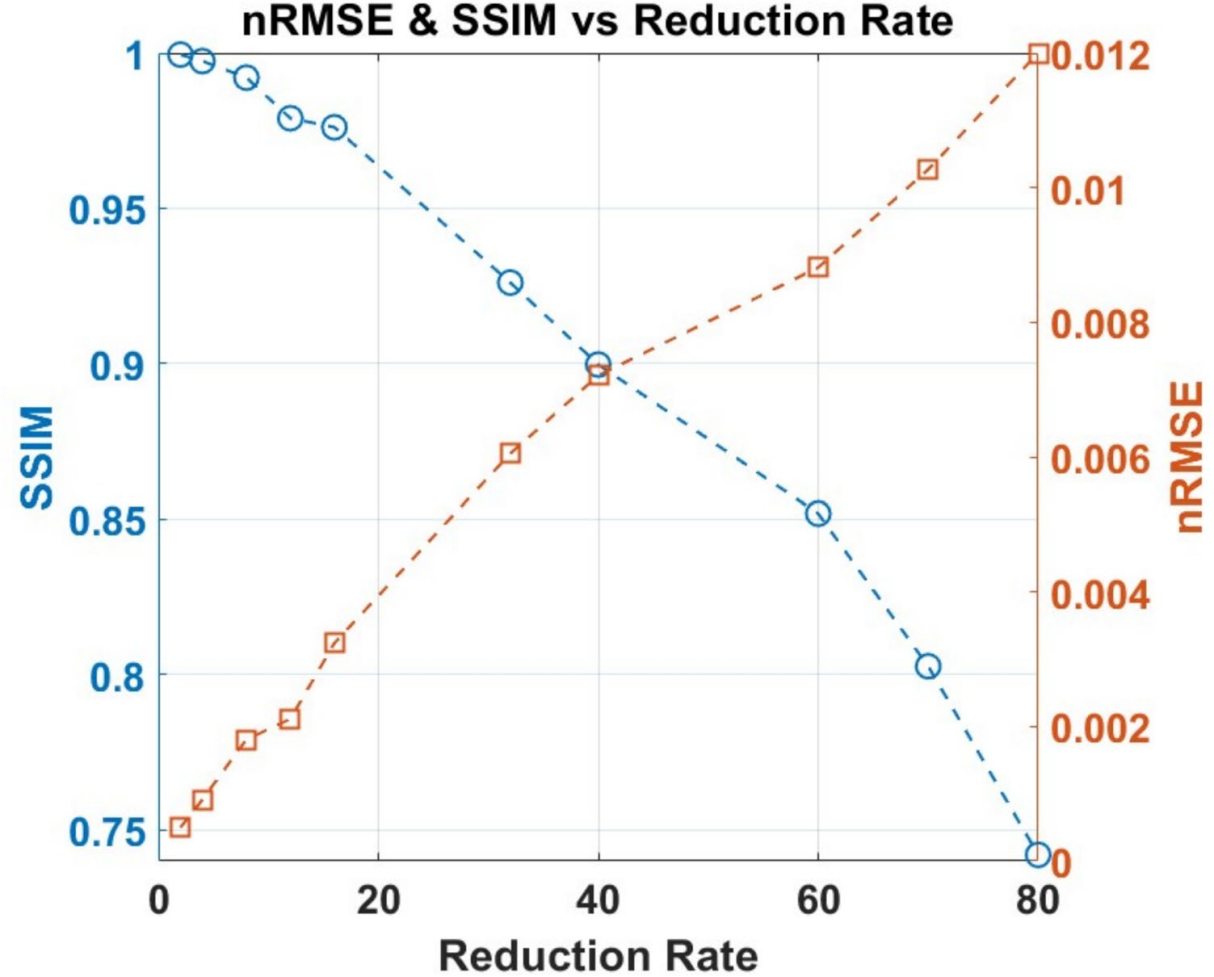
Relationship between reduction rate and reconstruction quality, measured by normalized root-mean-square error (nRMSE) and structural similarity index (SSIM). For reduction rates up to *R* = 16, equivalent to a 6.25% sampling rate, nRMSE remains very low (~ 0.003) and SSIM remains high (~ 0.977), indicating excellent fidelity. At higher reduction rates, artifacts become progressively more pronounced. Even at *R* = 80, corresponding to a 1.25% sampling rate, the reconstructed image remains recognizable despite visible artifacts (see Fig. 4)

Performance evaluation was conducted using two quantitative metrics: the normalized root-mean-square error (nRMSE) and the structural similarity index (SSIM), as described in Section B.2. The results, summarized in Fig. 3, demonstrate excellent reconstruction quality. Up to reduction rate *R* = 16, the nRMSE remained below 2 × 10⁻^3^, while SSIM consistently exceeded 0.97, reflecting the high fidelity of the compressed sensing (CS) reconstruction enabled by the proposed algorithm.

A key objective was to achieve a 16-fold reduction in acquisition time (*R* = 16) to enable real-time 3D-MRSI. At this reduction rate, the reconstruction yielded an nRMSE of 2.336 × 10⁻^3^ and an SSIM of 0.977, both indicating minimal deviation from the fully sampled reference.

**Fig. 4:**
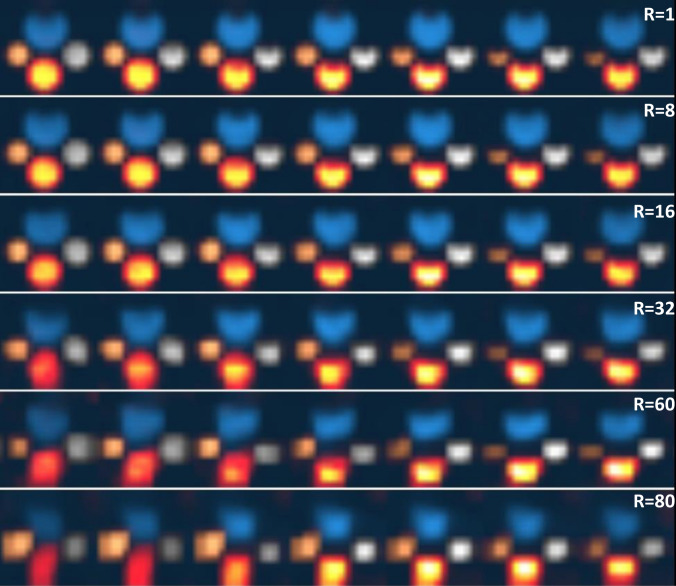
Reconstructed 3D-MRSI phantom images with a matrix size of 16 × 16 × 16. The central 8 of 16 slices are shown for each reduction rate, arranged from top to bottom rows as follows: *R* = 1 (100% sampling), *R* = 8 (12.5%), *R* = 16 (6.25%), *R* = 32 (3.125%), *R* = 60 (1.67%), and *R* = 80 (1.25%). Four cylinders containing partially vaporized chemicals—bicarbonate (top, blue), lactate (right, white), formate (bottom, yellow and red), and alanine (left, gold)—are overlaid on each image

Figure 4 displays the reconstructed 3D-MRSI metabolite maps for reduction factors *R* = 1 (fully sampled), *R* = 8, *R* = 16, as well as higher reduction rates *R* = 32, *R* = 60, and *R* = 80. Four cylinders with chemicals partially vaporized, bicarbonate, lactate, formate, and alanine, are overlaid on the same image using different colormaps. The metabolite distributions of [1-^13^C]lactate, [1-^13^C]alanine, [1-^13^C]formate, and [1-^13^C]bicarbonate remain visually consistent with the fully sampled data up to *R* = 16. Minor noise becomes apparent at *R* = 32 (3.125% sampling), yet the overall image quality and structural integrity remain well preserved, demonstrating the robustness of the CS reconstruction even at high undersampling rates. Additional phantom experiments at acceleration factors of *R* = 40, 50, 60, 70, and 80 (down to 1.25% sampling) showed progressively severe artifacts beyond *R* = 16, with *R* = 80 images losing coherent structure. These results establish *R* = 16 as a practical upper limit for high-fidelity reconstruction in our implementation.

B. In vivo rat kidney imaging

**Fig. 5:**
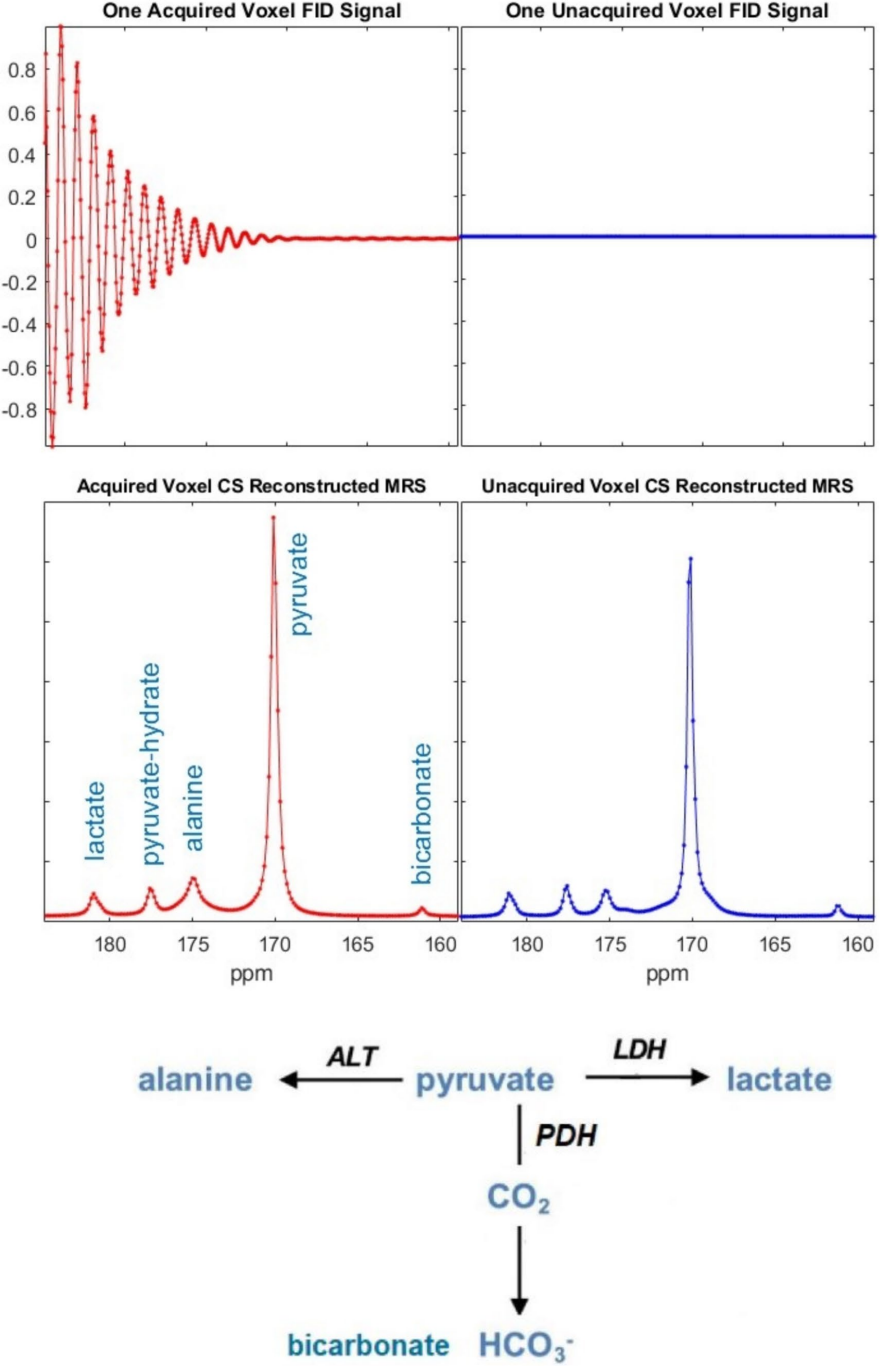
Demonstration of the effectiveness of the 4D-CS reconstruction from an in vivo rat scan with an acceleration factor of *R* = 16. The left column (in red) presents the acquired free induction decay (FID) and its corresponding spectrum from a voxel near the center of k-space. The right column (in blue) shows the originally unacquired FID (initialized as zeros) and the spectrum reconstructed by the 4D-CS algorithm from an adjacent voxel. The diagram illustrates the in vivo metabolic pathway of injected hyperpolarized [1-^13^C]pyruvate and the resulting in vivo production of metabolites, including lactate, alanine, and bicarbonate

An illustration of effective spectral reconstruction using the proposed 4D compressed sensing (CS) approach is shown in Fig. 5. This figure presents the acquired free induction decay (FID) of a voxel near the center of 3D k-space alongside a neighboring voxel for which no signal was acquired (zero-filled), and their corresponding spectra after CS reconstruction. These voxels were randomly selected from an in vivo rat kidney dataset acquired with a reduction factor of *R* = 16, where only 256 out of 4096 spatial points (6.25%) were sampled.

This example demonstrates the ability of the 4D-CS algorithm to accurately reconstruct spectral information even in the absence of direct signal acquisition. The reconstructed spectra preserve key metabolite peaks with high fidelity, highlighting the robustness of the CS framework for sparse MRSI. This capability is particularly valuable in hyperpolarized ^13^C-MRSI, where rapid signal decay and limited dose availability make efficient data acquisition essential.

**Fig. 6:**
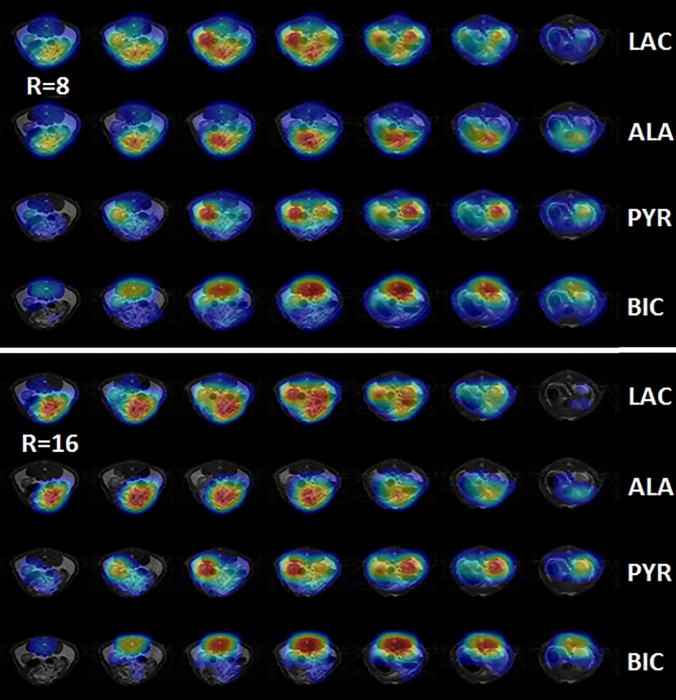
Selected slices from the reconstructed in vivo 3D-MRSI metabolic images of a rat kidney sectin. The top panel shows data acquired at a reduction rate of *R* = 8 (12.5% sampling), while the bottom panel shows data from *R* = 16 (6.25% sampling). Each panel is organized into four rows: lactate, alanine, pyruvate, and bicarbonate (from top to bottom). Different metabolites are localized in distinct tissue regions

Figure 6 presents reconstructed 2D metabolic maps of lactate, alanine, pyruvate, and bicarbonate from a rat kidney section acquired using undersampling rates of *R* = 8 and *R* = 16. These slice-wise images capture the spatial distribution of metabolites within the kidney, demonstrating the effectiveness of the 4D-CS method across varying sampling levels.

**Fig. 7:**
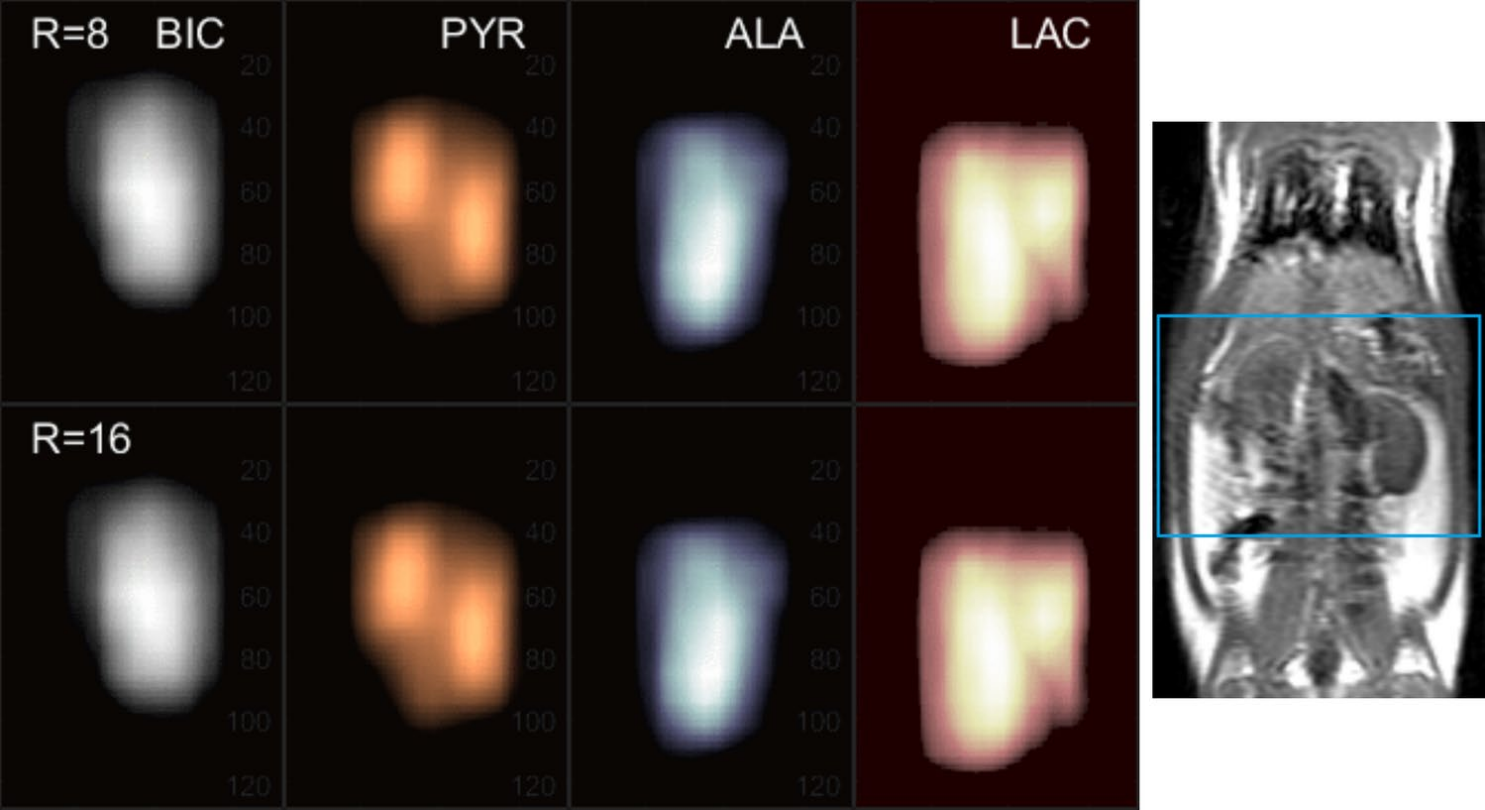
Animated Maximum-Intensity-Projection (MIP) reconstructed 3D-MRSI of different metabolites from the rat kidney section. From left to right: bicarbonate (BIC), pyruvate (PYR), alanine (ALA), and lactate (LAC). The top row is from *R* = 8 with 12.5% sampled data and the bottom row is from R = 16 with 6.25% sampled data. The image on the right shows the anatomical proton image and the region of MRSI

To enhance spatial interpretation, Fig. 7 also includes 3D maximum intensity projection (MIP) views for the same metabolites. These MIPs consolidate signal intensities across the full volume, revealing metabolic patterns more clearly and enabling better visualization of regional distributions. The combination of 2D slices and 3D MIPs underscores the consistency and reliability of the CS reconstruction, even at high acceleration factors.

These imaging results validate the in vivo metabolic activity following the injection of hyperpolarized [1-^13^C]pyruvate. Pyruvate is rapidly delivered to and concentrated in the kidneys via tail vein injection, where it undergoes enzymatic conversion into downstream metabolites. Lactate is primarily localized within the kidneys, while alanine and bicarbonate exhibit broader distribution in adjacent tissues. This spatially resolved mapping offers direct insight into organ-specific metabolic processes.

## Discussion

This study demonstrates that applying compressed sensing (CS) within a unified four-dimensional (4D) optimization framework enables highly efficient reconstruction of sparsely sampled MRSI datasets. By treating the entire 4D dataset as a single entity, this approach achieves high-quality reconstructions even at substantial acceleration factors, such as *R* = 16. This represents a significant improvement over conventional 3D-MRSI, which requires thousands of excitations and lengthy acquisition times, limiting its routine use in preclinical research. It is important to note that SSIM measures overall structural similarity, which can remain high even if fine details are lost. Therefore, in CS reconstruction, truly high-quality results typically exhibit SSIM values > 0.95. Even with only 1.25% of the data acquired (*R* = 80), despite the presence of artifacts, the reconstructed image remains recognizable. This underscores the superior performance of the unity compressed sensing method compared to slice-based reconstruction approaches.

The ability to dramatically reduce the number of excitations opens the door for practical, time-efficient imaging, particularly in applications involving hyperpolarized ^13^C-labeled compounds, where magnetization is non-renewable. The conventional conjugate gradient method, applied here within the 4D-CS framework, proves well suited for reconstructing undersampled 3D-MRSI data, delivering results with minimal loss of fidelity.

During algorithm tuning, it was observed that relying solely on intermediate variable convergence to terminate optimization may be suboptimal. The convergence curve often progresses in steps, and similar intermediate states can mask ongoing changes in data structure. Premature termination may prevent reaching the true minimum. Therefore, both stopping thresholds (ε and ϵ, defined in Section II.B) must be set sufficiently low to ensure full convergence.

Minor differences observed between the *R* = 8 and *R* = 16 rat metabolite images could stem from the increased undersampling at *R* = 16 or from physiological variability introduced by the second injection of hyperpolarized pyruvate in the same animal. The resulting metabolite distributions, pyruvate mainly in the kidneys, lactate in the kidneys and intestine, alanine in the intestine, and bicarbonate in the back muscle, are consistent with known metabolic pathways, though further validation in collaboration with biomedical researchers is warranted.

Crucially, this 4D-CS method preserves the intrinsic spatial and spectral coherence of the full dataset, in contrast to previously reported slice-by-slice or sequential reconstructions, which can lead to discontinuities and degraded spectral quality. By exploiting the natural compressibility of the full 4D data, this approach supports accurate 3D-MRSI in both preclinical and clinical settings.

In this work, we successfully reduced the excitation count for a 16 × 16 × 16 MRSI matrix from 4096 to just 256 (a 16-fold reduction), a level consistent with acquisition times used in 2D hyperpolarized ^13^C-MRSI studies [12–14]. The combination of our 4D optimization strategy and careful parameter tuning ensures robust convergence and avoids entrapment in local minima, ultimately delivering reliable and high-quality reconstructions suitable for real-time MRSI. Additional information is provided in the ‘Supplementary Material’ file.

## Supplementary Information

Below is the link to the electronic supplementary material.

Supplementary file1 (DOCX 9206 kb)

## Data Availability

Data will be made available on reasonable request.

## Abbreviations

MRI: Magnetic resonance imaging
MRSI: Magnetic resonance spectroscopic imaging
CSI: Chemical shift imaging
3D/4D: Three dimensional/four dimensional
RF: Radio-frequency
EPSI: Echo-planar spectroscopic imaging
nRMSE: Normalized root-mean-square error
SSIM: Structural similarity index
FID: Free-induced-decay
HP: Hyperpolarized/hyperpolarization
CS: Compressed sensing
BW: Bandwidth
MIP: Maximum intensity projection
