# Supplementary material for: Fast 3D-MRSI using sparse acquisition and 4D compressed sensing reconstruction
Source: MAGMA. 2025 Nov 1;39(2):253–64. doi: 10.1007/s10334-025-01301-y (PMC13124777; doi:10.1007/s10334-025-01301-y)
Supplement: Supplementary file 1 — Supplementary file1 (DOCX 9206 kb) [file 10334_2025_1301_MOESM1_ESM.docx]

Symbolic Representation of 3D Wavelet:


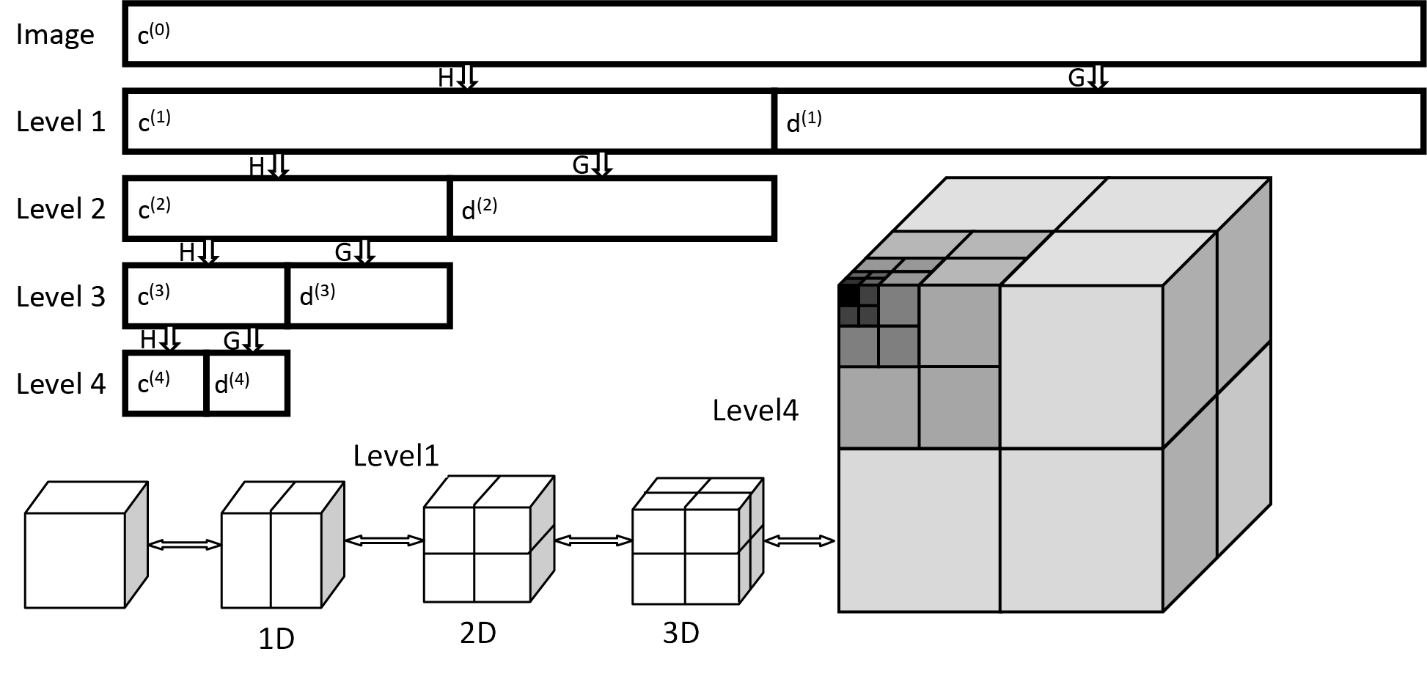


Symbolic Representation of 4D Wavelet:


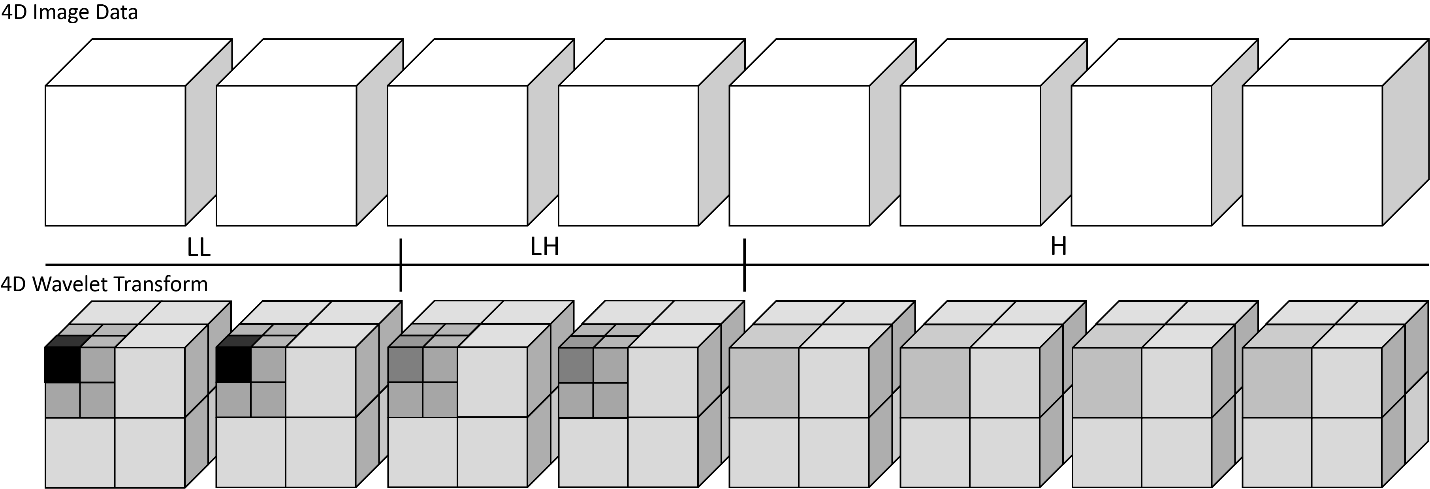


4D Unity Compressed Sensing reconstructed phantom spectra:


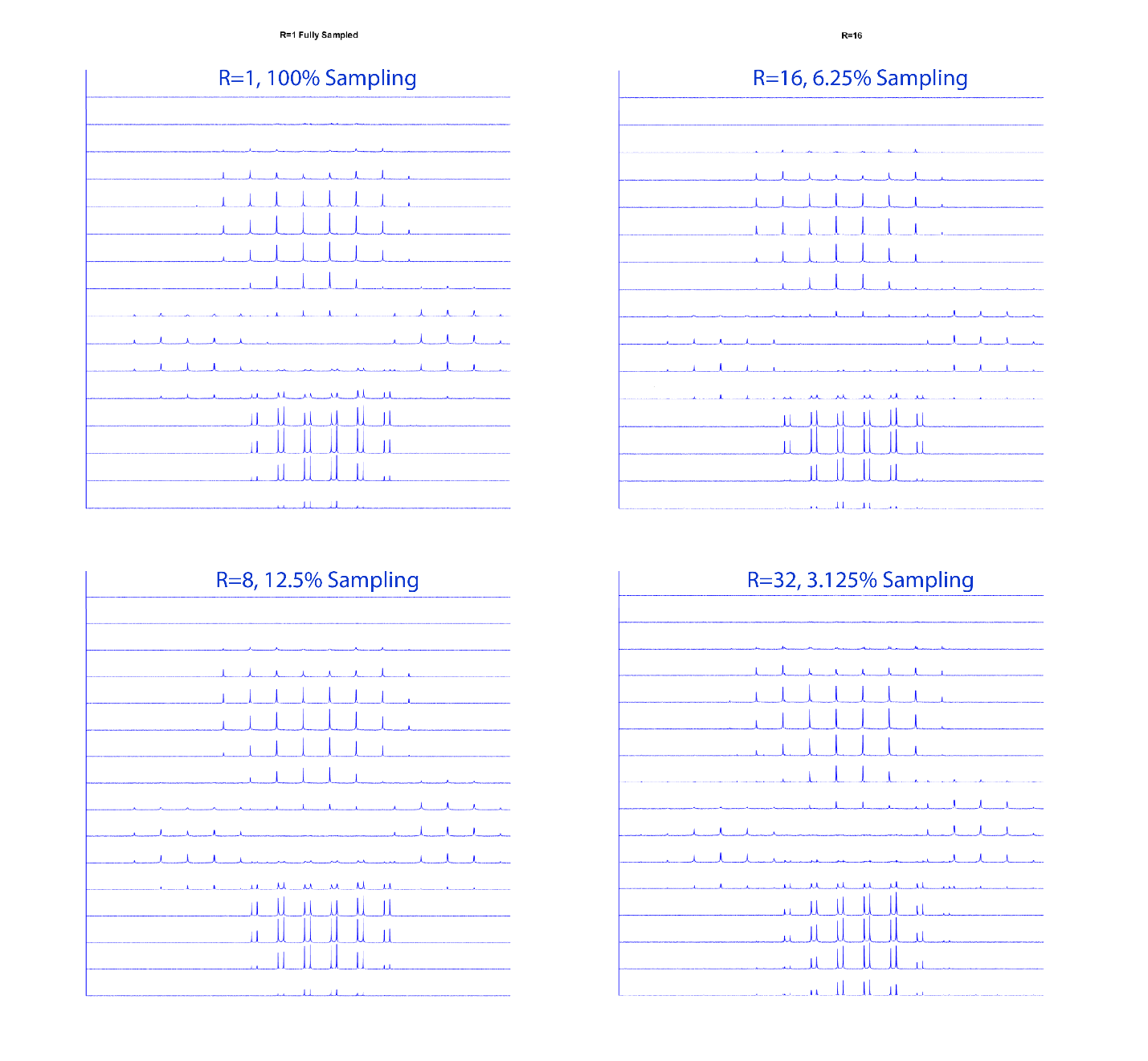


Additional phantom spectra reconstructed using 4D Unity Compressed Sensing from sparsely sampled voxels. Sparsity constraints were applied in three spatial dimensions, although only a single slice is displayed here.
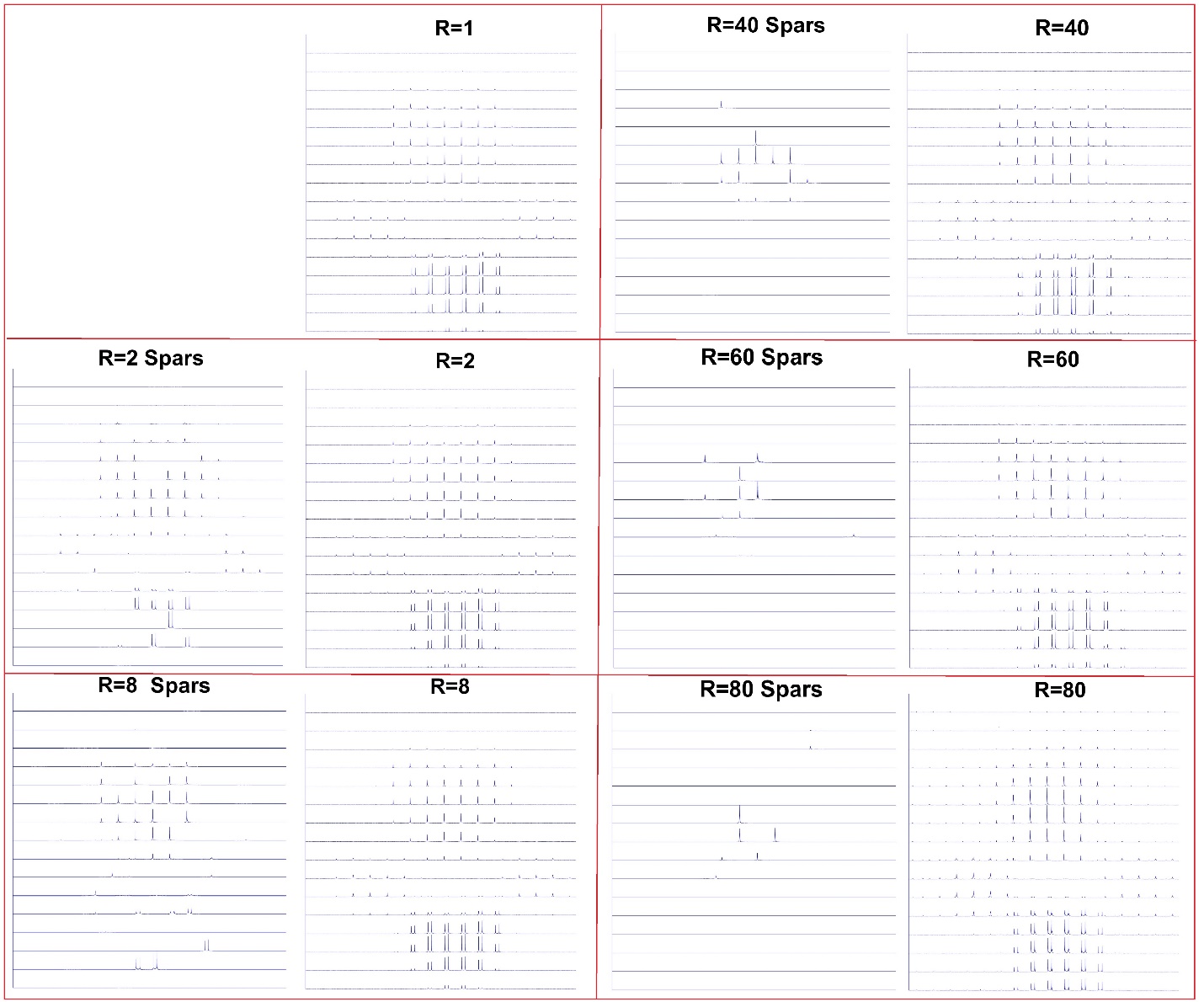


Rat heart has also been imaged with the same method:


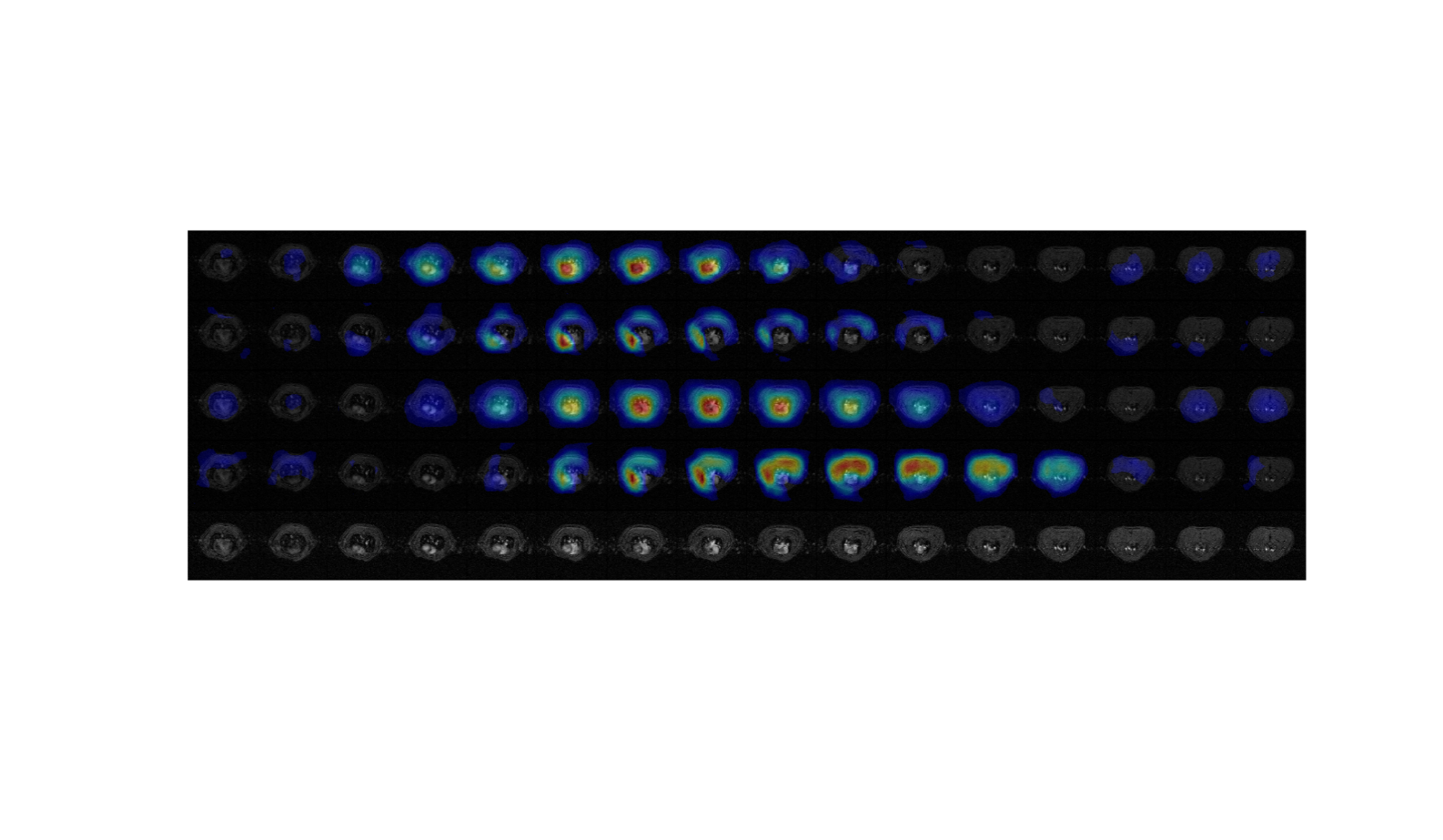

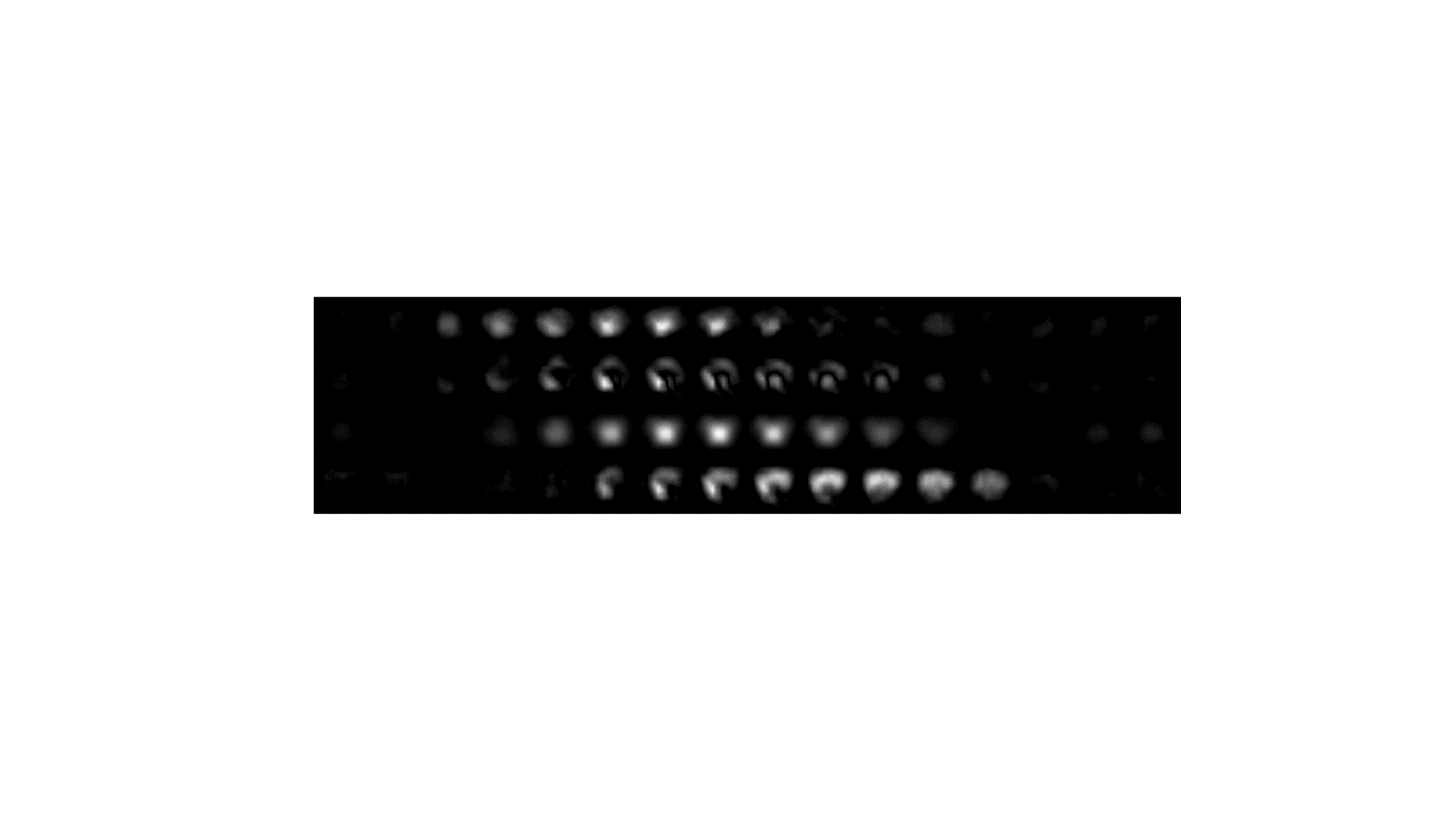


lactate

alanine

pyruvate

bicarbonate

lactate

alanine

pyruvate

bicarbonate

GIF file showing the animated sequence of sparse acquisition for an N³-voxel dataset with a reduction factor of 16.


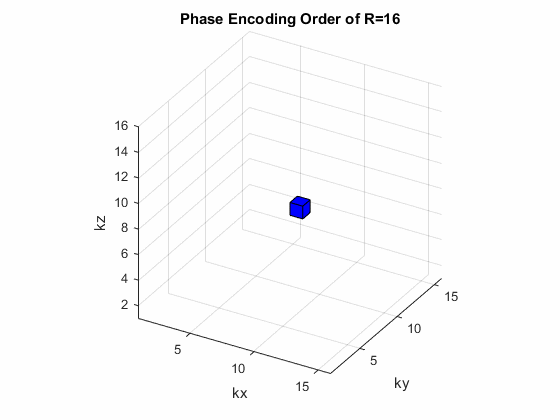


GIF file showing animated in vivo rat kidney MIP images following injection of [1-¹³C]pyruvate and its metabolic products, bicarbonate, alanine, and lactate, reconstructed with reduction factors of 8 and 16, respectively.


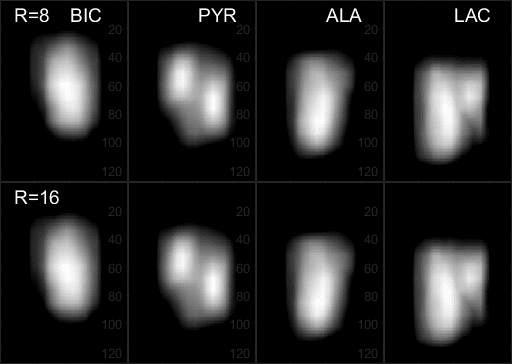


lactate

alanine

pyruvate

bicarbonate

lactate

alanine

pyruvate

bicarbonate
